# Associations between Indoor Environmental Quality and Infectious Diseases Knowledge, Beliefs, and Practices of Hotel Workers in Wuhan, China

**DOI:** 10.3390/ijerph18126367

**Published:** 2021-06-11

**Authors:** Wenjing Wang, Yixin Liu, Ling Zhang, Li Ran, Siyuan Xiong, Xiaodong Tan

**Affiliations:** 1 School of Health, Wuhan University, Wuhan 430071, China; Wangwj96@whu.edu.cn (W.W.); zhangling0@whu.edu.cn (L.Z.); 2019103050005@whu.edu.cn (L.R.); 2 Hongshan Center for Disease Control and Prevention, Wuhan 430071, China; lyx18086532528@163.com (Y.L.); Bear.yes@163.com (S.X.)

**Keywords:** knowledge, beliefs, practices, environmental quality, infectious diseases

## Abstract

Knowledge, beliefs, and practices regarding infectious diseases are key elements that ensure practitioners’ health and safety. It is important to carry out such a survey in hotels. This study aims to determine the levels of knowledge, beliefs, and practices regarding infectious diseases among practitioners and their associations with the environmental quality of hotels in Wuhan, China. We surveyed infectious disease knowledge, beliefs, and practices of practitioners in 18 hotels and detected these hotels’ environment, including physical factors of temperature, humidity, noise, and the indoor air quality of benzene, toluene, xylene, formaldehyde, CO, CO_2_, the total count of fungi, aerobic plate count, PM_10_, and PM_2.5_. 128 practitioners were included, and 28.9% were male. The questionnaire included knowledge, beliefs, and practices regarding infectious diseases. Our study found moderate levels of knowledge and beliefs, and good health practices. People’s beliefs toward COVID-19 were correlated significantly with their knowledge (*p* < 0.05). Beliefs and health practices were correlated significantly with environmental quality (*p* < 0.05). However, the environmental quality was correlated negatively with the classification of hotels. Conclusively, despite the good health practices of practitioners, the knowledge and beliefs toward infectious diseases need to strengthen. Hotels should emphasize health education in practitioners and the improvement of environmental hygiene. Integrating all three components into a comprehensive environmental promotion program is warranted.

## 1. Introduction

Public places facilitate people to carry out a variety of social activities, and after the pandemic of Coronavirus Disease 2019 (COVID-19), the public has paid more attention to health security in public places [1]. However, there are multiple hygienic problems in hotels. Characterized by a lack of sanitation, significant passenger loads and reused public appliances, the poor environment of hotels is prone to result in the spread of diseases and cross-contamination [2,3]. In China, hotels of the top 10 cities have a total of 70,078 and an average of 7000 hotels per city [4]. Therefore, the disinfection and cleaning guidelines in hotels play a vital role in people’s health [5].

Environmental quality should be concerned in many respects. A study in the designated hospital for COVID-19 suggests that the environment is a potential medium of disease transmission, and the need for strict environmental surface hygiene in order to prevent the spread of the virus is emphasized [6]. In Jiangsu Province, China, a patient with SARS-CoV-2 may have transmitted the virus to eight other healthy individuals via bathing in a public bath center [7]. The results of a cohort study among visitors of a hotel suggest that environmental contamination should be considered as a possible source of infection [8]. These results emphasize the importance of environmental hygiene and the necessity of improving environmental quality. In hotels, priority is given to hazardous factors that pose a greater risk, occur more frequently, have a heavier environmental load, and are of higher public concern. Meanwhile, the ease of organization and operability should be considered. Therefore, physical factors (temperature, humidity, noise) and indoor air quality (benzene, toluene, xylene, formaldehyde, CO, CO_2_, the total count of fungi, aerobic plate count, PM_10_, and PM_2.5_) are used to represent the environmental quality of hotels. The provision of good sanitation and hygiene conditions is essential for protecting human health in outbreaks of infectious diseases, including COVID-19.

Most of the people surveyed are healthcare workers. Few studies have been carried out among workers of hotels [9,10,11,12]. However, many hotel workers are at high risk for exposure to occupational stress [13]. These stressors include low job security, inadequate training, and work environment disparities [14,15,16]. Considering the close and frequent contact with other individuals, workers can be exposed to respiratory and gastrointestinal infections through a variety of mechanisms including sneezing, coughing, and touching fomites within the workplace [17,18]. Knowledge of infectious diseases and personal health practices could improve the health of workers and assist with the control of such outbreaks. The workplace is an important venue for efforts to reduce the impact of infectious diseases. In turn, workers who have good infectious disease knowledge, beliefs, and practices can better protect themselves and maintain a clean work environment [19]. Moreover, several studies evaluate knowledge, beliefs, and practices toward other aspects, such as food safety, sanitation hygiene, and home environment prevention. The results show that a high level of knowledge, beliefs, and practices is beneficial [20,21,22].

This study aims to evaluate the association between knowledge, beliefs, and practices toward the prevention and control of infectious diseases and environmental quality among practitioners of hotels in Wuhan City in 2020. Such studies are rare; to make up the gap and better improve the environmental quality of hotels, we conduct this study in Wuhan, once the hardest-hit area.

## 2. Subjects and Methods

### 2.1. Study Design

The National Health and Family Planning Commission issued the National Pilot Program for monitoring health hazard factors in public places in 2016. Wuhan, as one of the four pilot cities in Hubei Province of China, has carried out monitoring work every year. The ethical review of the protocol has been approved by the Ethics Committee of the School of Medicine, Wuhan University (2021YF0052). This study investigated three parts: (1) the basic situation of the practitioners; (2) the knowledge, beliefs and practices of the practitioners, (3) and the environmental quality of hotel. We monitored 18 hotels from October to December, including six chain hotels, six hotels below three-star ratings and six hotels above three-star ratings. In each category of hotels, we first sorted them according to the opening time, and then numbered them sequentially. Finally, a random sampling method was used to choose these hotels.

### 2.2. Participants

A total of 128 subjects were selected, consisting of 23 practitioners from chain hotels, 45 from the hotels below three stars, and 60 from the hotels above three stars. Face-to-face interviews were conducted to collect information from the participants, who were informed of the project details and signed an informed consent. All these investigators were trained on the research purpose, survey procedure, and other important matters before conducting the survey. The questionnaire, compiled by professionals in the field of environmental hygiene in China, includes basic information as well as knowledge, beliefs and practices regarding the prevention and control of infectious diseases.

The practitioners’ ages were determined from the records on their identification cards. The sex, length of service, daily working hours, smoking and drink history, and educational background were self-declared.

### 2.3. Knowledge, Beliefs, and Practices Regarding the Prevention and Control of Infectious Diseases

The knowledge questionnaire consisted of three items on whether the hotel’s public appliances spread diseases, whether the participants know the classification of infectious diseases and the main routes of transmission of COVID-19 (respiratory transmission and contact transmission). The full marks of the knowledge part are eight points.

The beliefs questionnaire covered four items, including disinfection method (excellent, average, poor, none), measures for the prevention and control of infectious diseases (wear masks, wash hands frequently, gather less, ventilate more and I don’t know), situations that don’t need to wear masks (at home and outdoors, at home or outdoors, wrong), and excessive disinfection (disinfect the pond, disinfect the external environment air, and spray disinfection on the whole body of person, wrong). Full marks for the beliefs part is 12 points.

The practices questionnaire contains seven items about healthy behavior which are whether to actively apply for a health certificate, whether to wash their hands with hand sanitizer, whether to wear work clothes, whether to wear protective gloves, whether to wear protective masks, whether to go to work when coughing or with a fever, and whether to avoid people when coughing or sneezing. The subjects answered “true” or “false” for each item. A correct answer was given one point, and an incorrect answer was given zero points. Full marks for the practices part is seven points.

### 2.4. Environmental Quality of the Hotel

The compliance rate of physical factors and the indoor air quality was monitored to represent the environmental quality of the hotel. The physical factors included temperature, humidity, noise, and the indoor air quality included benzene, toluene, xylene, formaldehyde, CO, CO_2_, the total count of fungi, aerobic plate count, PM_10_, and PM_2.5_. 

#### 2.4.1. Overview of Environmental Monitoring Indicators

All environmental indicators were measured and recorded using different methods (Table 1), and then collated and compared with the National Standards of the People’s Republic of China (GB 37488-2019: hygienic index and limit requirements of public places; JGJ/T 309-2013: test and evaluation standard of ventilation effect of buildings). The categories of monitoring indicators, sampling environment, sampling quantity and so on are detailed in Table 1. Items that meet the standards were given one point, and those that fail were given zero points. Full marks for the environmental quality is 13 points.

#### 2.4.2. Sampling Location Distribution of the Hotel

**Figure 1:**
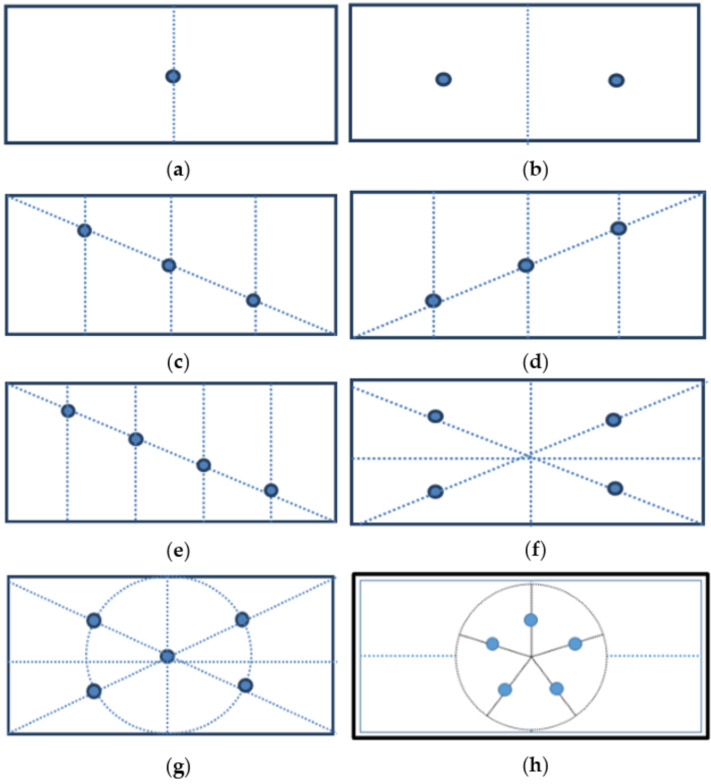
The setting of sampling points. Note: (**a**): 1 sampling point, (**b**): 2 sampling points, (**c**): 3 sampling points, (**d**): 3 sampling points, (**e**): 4 sampling points, (**f**): 4 sampling points, (**g**): 5 sampling points, (**h**): 5 sampling points.

Above all, the environment required the doors and windows to be closed before sampling. The location of sampling points be set in accordance with the principle of uniform distribution, specifically based on the number of sampling points, as follows. One sampling point: set in the center (Figure 1a); two sampling points: set on the symmetry point (Figure 1b); three sampling points: set on three equal points of diagonal quadrants, as shown in Figure 1c,d; four sampling points: set on the four equal points in five equal parts of the diagonal (Figure 1e), or on the center of the four equal areas (Figure 1f); five sampling points: the cross plum distribution point (Figure 1g) or dog claw plum distribution point (Figure 1h). When monitoring noise, it should be set at three equinox points of a straight line quadripartite from the center of the noise source to the center of the farthest wall on the opposite side.

### 2.5. Statistical Analysis

Statistical analysis was performed by the use of Statistical Package for Social Science Version 22 (IBM, Armonk, NY, USA). Statistical significance was set as *p* < 0.05. Continuous variables were expressed as mean (standard deviation), while categorical variables were expressed as count and percentage. The chi-square tests were used to compare the rate in different groups and to choose variables for the correlation analysis. Principal component analysis (PCA) was used to reduce the number of variables in a multivariate data set, and it can retain the variation present in the data set. Pearson correlation was used to identify the relationship among knowledge, beliefs, and practices with the environmental quality of the hotel.

## 3. Results

### 3.1. Sociodemographic Characteristics of Subjects

A total of 128 subjects, comprising 37 men and 91 women, were enrolled in the study. Ages from 40 to 59 years were the most common, accounting for 46.9%. The majority of the practitioners had worked in hotels for 1–5 years and 93% of them worked ≤8 h per day. Most of them people did not smoke or drink. When it comes to educational background, the majority of the practitioners were junior college or undergraduates.

### 3.2. Level of Knowledge, Beliefs, and Practices Regarding the Prevention and Control of Infectious Diseases

As shown in Table 2, approximately more than 85% of practitioners knew whether the hotel’s public appliances spread diseases. The classification of infectious diseases and the transmission route of COVID-19 were, respectively, mastered by 75.8% and 71.1% of practitioners. In the knowledge score, 47.7% of practitioners achieved full marks.

Ninety-three percent of practitioners believed that wearing masks, washing hands frequently, gathering less, and ventilating more are beneficial in preventing and controling COVID-19; 78.1% thought it unnecessary to wear masks at home and outdoors, and 64.9% chose spray disinfection, wiping or soaking to disinfect public places. Only 6.3% of practitioners understood what over-disinfection is, and 9.4% even thought that disinfection in frequently touched places was excessive. In the beliefs score, 71.9% of practitioners achieved 10 points or more.

Among the health practices of practitioners, each person wore a mask, and more than 90% of them actively applied for health certificates, washed their hands with hand sanitizer, wore work clothes, and so on. When practitioners worked, 83.6% of them wore protective gloves. Practices score showed that 95% of practitioners achieved 6 points or more.

Overall, in the total score, no one achieved full marks; the majority of practitioners scored 23 points or above, accounting for 75.8%, and the mean total score was 23.4 (SD = 2.67). 

### 3.3. Environmental Quality

A total of 816 samples were monitored in this study. Among them, there were 180 samples of physical factors and 636 samples of indoor air. The total compliance rate of hotels was 78.6%. In monitoring indicators, the values of noise, benzene, toluene, xylene, formaldehyde, CO, CO_2_, and aerobic plate count in hotels were up to the standard. The compliance rates of temperature, humidity, and PM_10_ in the hotels were 50%, 65%, and 66.7% respectively. However, the compliance rate of the total count of fungi was 36.7%, and the PM_2.5_ was 23.1%.

**Figure 2:**
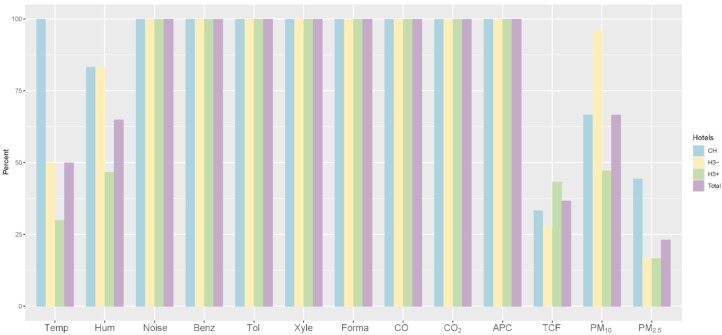
The compliance of the environmental quality of hotels. Note: Temp (temperature), Hum (humidity), Benz (benzene), Tol (toluene), Xyle (xylene), Forma (formaldehyde), APC (aerobic plate count), TCF (total count of fungi). CH (chain hotels), H3− (hotels below 3-star), H3+ (hotels above 3-star).

On the whole, 3.9% of hotels met all environmental standards. Four in 10 hotels had a score of 10 points for environmental quality, with 25% scoring above 10. The average points of chain hotels, hotels below three stars and hotels above three stars were 11.96, 11.31 and 10.33, respectively. The total score of the environmental quality of hotels was 11.2. It is worth noting that the hotels above three stars achieved the lowest scores, while the chain hotel achieved the highest scores in this environmental monitoring. Details are shown in Figure 2.

### 3.4. Group Comparison in Environmental Quality

The chi-square test results showed that environmental quality score was statistically significant for whether towels (*p* < 0.001), slippers (*p* < 0.001), bathtubs (*p* < 0.001), teacups (*p* < 0.001) and toilets (*p* < 0.001) spread disease. Statistical significance was also observed for knowing the classification of infectious diseases (*p* < 0.001), main routes of transmission of COVID-19 (*p* < 0.001), whether there was a need to wear masks (*p* < 0.001), and the question regarding whether to go to work when coughing or with a fever (*p* = 0.01).

### 3.5. Principal Component Analysis (PCA)

Considering that the statistically significant variables have collinearity with each other, PCA was used to reclassify these variables, and the new variables obtained through dimensionality reduction can better reflect the relationship with environmental quality. The results of PCA showed that KMO > 0.6, *p* < 0.05 in Bartlett test. The first PC (PC1) correlated significantly with the content of whether teacups, towels, slippers, toilets, and bathtubs spread disease. Thus, PC1 substantially described whether the hotel’s public appliances spread diseases. The second PC (PC2) also correlated significantly with the content of the classification of infectious diseases and the situations that don’t need to wear masks. Thus, PC2 substantially described the basic knowledge of infectious diseases. Furthermore, PC3 substantially described the routes of spreading infectious diseases.

### 3.6. Correlation between Knowledge, Beliefs, Practices, and Environmental Quality

This study demonstrated that knowledge was correlated with several aspects of beliefs toward infectious diseases and health practices. A positive correlation was noted between knowledge score and beliefs score (*r* = 0.224, *p* = 0.011), total score (*r* = 0.830, *p* < 0.001), the classification of hotel (*r* = 0.210, *p* = 0.017), whether the hotel’s public appliances spread diseases (*r* = 0.942, *p* < 0.001), basic knowledge of infectious diseases (*r* = 0.197, *p* = 0.026), as well as understand the routes of spreading infectious diseases (*r* = 0.184, *p* = 0.038). Beliefs score regarding infectious diseases correlated significantly with several aspects, including total score (*r* = 0.686, *p* < 0.001), the classification of hotel (*r* = 0.267, *p* = 0.002), and the basic knowledge of infectious diseases (*r* = 0.587, *p* < 0.001). In other words, a negative relationship was found between beliefs score and environmental quality score (*r* = −0.198, *p* = 0.025). In addition, practices score was associated with total score (*r* = 0.307, *p* < 0.001), environmental quality score (*r* = −0.177, *p* = 0.046), and understanding of the routes of spreading infectious diseases (*r* = 0.306, *p* < 0.001). Additionally, total score was also associated with the classification of hotel (*r* = 0.258, *p* = 0.003), whether the hotel’s public appliances spread diseases (*r* = 0.726, *p* < 0.001), basic knowledge of infectious diseases (*r* = 0.444, *p* < 0.001), and understanding of the routes of spreading infectious diseases (*r* = 0.193, *p* = 0.029). A positive relationship was indicated between understanding of the routes of spreading infectious diseases (*r* = 0.184, *p* = 0.038) and environmental quality score. A negative relationship between environmental quality score and the classification of hotel (*r* = −0.543, *p* < 0.001), basic knowledge of infectious diseases (*r* = −0.387, *p* < 0.001) was also noted (Table 3).

## 4. Discussion

The current study was conducted among practitioners in hotels where environmental quality is vital for people’s health. Most studies concern healthcare workers, due to their health being closely related to their working environment [23,24]. However, targeting the practitioners of hotels is no less essential than healthcare workers for developing an effective intervention plan in public places and proposing an effective policy.

The participants had a moderate level of knowledge about infectious diseases. This finding was higher than a study in Italy (24.2%) [25]. However, this finding was lower than studies in China and Ethiopia [26,27]. The discrepancies may be due to differences in socio-demographic factors, such as educational status, sample size, and study population. The majority of practitioners can master the knowledge of whether the hotel’s public appliances spread diseases. However, knowledge of the classification of infectious diseases and the transmission route of COVID-19 mastered by practitioners need to improve. Most medical students (92.9%) knew the transmission route of COVID-19, according to a survey in Iranian [28]. The possible reason for the disparity may be that most of the practitioners were over 40 years old, and they had not had enough access to newspapers and the internet. This reflects limited information about infectious disease updates on preventive measures from government officials, social media, and the internet.

Most practitioners recognized the importance of wearing masks, hand-washing, ventilating, and less gathering in reducing the chances of contracting diseases. This finding was consistent with a bi-national survey in Africa [29]. The majority of them had a good understanding of over-disinfection and 9.4% had a wrong perception that disinfection infrequently touched places was excessive. However, the best way to prevent the spread of the virus in the environment was to encourage cleaning and disinfection in places and surfaces that were touched very frequently [30].

Among the practitioners, 95% had a high level of performance in preventive behaviors, which was higher than the previous studies [31,32]. All participants said that they wear masks when working. The feasibility and effectiveness of mask-wearing have been verified in previous researches. It is beneficial for both health workers and students [33]. However, one in five (16.4%) said that they will not wear protective gloves when working. One study showed that environmental control measures need to be applied alongside adherence to hand hygiene among all personnel [34]. The result reflects that personal health-protective awareness needs to strengthen.

Compared with previous studies in schools and hotels, the environmental quality of hotels in Wuhan was at a good level [35,36]. In the various monitoring indicators, half of the measured values for temperature and humidity had met the standard. Some studies showed that the resistance of the virus on inanimate surfaces was influenced by the following factors: the type of surface, the temperature of the environment, and the relative humidity of the air [37,38,39]. Therefore, the temperature and humidity in the hotel should be constantly considered, in order to protect the health of workers and customers. The air pollution of PM_2.5_ and PM_10_ thresholds are 75 μg/m^3^ and 150 μg/m^3^, as proposed by the World Health Organization (WHO) [40]. Studies have shown that the incidence and mortality of the population were correlated with the mass concentration of atmospheric particulates, particularly with the concentration of particulate matter in the room [41]. However, in these samples, approximately 80% of the concentration of PM_2.5_ exceeded the standard value. Therefore, it is recommended that more effective measures such as air purification and regular monitoring should be taken to ensure the hotel’s air quality and people’s health.

This study also illustrated that knowledge was correlated with beliefs about infectious diseases. The higher the knowledge score, the higher the belief score. Rationally, beliefs depend on an individual’s perception of a problem and perception could be modified by knowledge about the disease [42]. However, neither of them had any correlation with practices, which was different from the findings in Malaysia [43]. Additionally, the associations between knowledge, beliefs, and practices of practitioners and environmental quality were also determined in this study. No significant correlation was found between environmental quality and knowledge within this study. This finding contradicted the observation obtained among 848 households in China, which revealed a positive influence of the knowledge of air pollution on residents [44]. However, beliefs about infectious diseases and health practices were correlated with environmental quality. A positive relationship was indicated between health practices and environmental quality. The higher the practices score, the higher the environmental quality score. This may be due to the fact that practitioners pay more attention to healthy behaviors and then they pay more attention to the environment of hotel when they work. The relevant study has shown that the awareness and actions of people may promote environmental disinfection [6]. In addition, a negative relationship was noted between environmental quality and the classification of hotels. In other words, the higher level of hotels, the lower the environmental quality score. Given that good environmental quality improves the hotel industry, and brings better development to the hotel [45], the hotel should put more emphasis on the environment to guarantee the health of staff and customers.

The present study should be interpreted within the context of its strength and limitations. It is limited by its sample size, which is insufficient. In addition, the long-term effects of knowledge, beliefs, and practices with respect to infectious diseases were not assessed longitudinally, due to the inherent nature of the cross-sectional design. In addition, some unknown and omitted confounding factors may exist in this study; instrumental variable analysis was used to control these confounding factors. Despite these limitations, it was one of the few studies that attempted to determine the association between knowledge, beliefs, and practices with regard to infectious diseases and environmental quality of hotels among practitioners. It not only complements the past studies, but also contributes to better environmental quality of the hotels.

## 5. Conclusions

In summary, our study shows that there is a moderate level of knowledge and beliefs regarding infectious diseases but good health practices among practitioners of hotels. Knowledge regarding infectious diseases was associated with beliefs in this study. No significant correlation was found between environmental quality and knowledge regarding infectious diseases in this study. In addition, beliefs about COVID-19 and health practices were correlated with environmental quality. However, it was noted that there is a negative relationship between environmental quality and the classification of hotels. These conclusions suggest that a future awareness campaign should focus more on health education in practitioners from all walks of life, to increase knowledge and strengthen beliefs with regard to health. The hotels should increase efforts to devote to the questions of environmental hygiene.

## Figures and Tables

**Table 1:** List of environmental monitoring indicators for hotels.

Category	Sampling Environment	Indicators	Sampling Quantity	Monitoring Method
physical factors	indoor air	temperature, humidity	A. sample size: number of rooms ≤100 (3–5%); >100 (1–3%) B. number of sampling points: area (m^2^): <50 (1 sampling point); 50–200 (2 sampling points); >200 (3–5 sampling points)	apparatus: digital thermometer and digital hygrometer. data: direct reading measurement
	indoor environment	noise	same as above	apparatus: digital sound level meter. data: direct reading measurement
indoor air quality	indoor air	benzene, toluene, xylene	same as above	apparatus: Tenax TA adsorbent tube
		formaldehyde	same as above	apparatus: bubble absorption tube, air sampling pump
		CO, CO_2_, PM_10_, and PM_2.5_.	same as above	apparatus: carbon monoxide non-dispersive infrared gas analyzer, carbon monoxide non-dispersive infrared gas analyzer, light scattering dust test instrument. data: direct reading measurement
		the total count of fungi, aerobic plate count,	A. sample size: number of rooms ≤100 (3–5%); >100 (1–3%) B. number of sampling points: area (m^2^): <50 (1 sampling point); 50–200 (2 sampling points); >200 (3–5 sampling points)	apparatus: six-stage sieve percussion microbial sampler

**Table 2:** Level of knowledge, beliefs, and practices towards the prevention and control of infectious diseases.

Aspects	Category/Range	%	Mean (SD ^1^)
Level of knowledge	0–8		6.75 (1.90)
Do towels spread disease?	No	14.8	
	Yes	85.2	
Do slippers spread disease?	No	15.6	
	Yes	84.4	
Do bathtubs spread disease?	No	12.5	
	Yes	87.5	
Do teacups spread disease?	No	14.1	
	Yes	85.9	
Do toilets spread disease?	No	14.1	
	Yes	85.9	
Are there 3 types of infectious diseases?	No	24.2	
	Yes	75.8	
Main routes of transmission of COVID-19	Wrong,	0.8	
	Know part,	28.1	
	Understand all	71.1	
Level of beliefs	0–12		10 (1.33)
Disinfection method	None	0	
	Poor	7	
	Average	28.1	
	Excellent	64.9	
Measures for the prevention and control of infectious diseases	I don’t know	0	
	Know 1 measures	0.8	
	2 = Know 2 measures	2.4	
	3 = Know 3 measures	3.9	
	4 = Understand all	92.9	
Situations that don’t need to wear masks	0 = Wrong	0.8	
	1 = At home or outdoors	21.1	
	2 = At home and outdoors	78.1	
Excessive disinfection	0 = Wrong	9.4	
	1 = Know 1	22.6	
	2 = Know 2	61.7	
	3 = Understand all	6.3	
Level of practices	0–7		6.66 (0.59)
Whether to actively apply for a health certificate?	0 = No	3.1	
	1 = Yes	96.9	
Whether to wash their hands with hand sanitizer?	0 = No	1.6	
	1 = Yes	98.4	
Whether to wear work clothes?	0 = No	4.7	
	1 = Yes	95.3	
Whether to wear protective gloves?	0 = No	16.4	
	1 = Yes	83.6	
Whether to wear protective masks?	0 = No	0	
	1 = Yes	100	
Whether to go to work when coughing or with a fever?	0 = Yes	6.3	
	1 = No	93.7	
Whether to avoid people when coughing or sneezing?	0 = No	2.3	
	1 = Yes	97.7	
Total score			23.4 (2.67)

^1^ SD: standard deviation.

**Table 3:** Correlation between knowledge, beliefs, practices of practitioners, and environmental quality (*n* = 128).

		A.	B.	C.	D.	E.	F.	G.	H.	I.
A. Practice score	*r*	-	0.028	0.130	0.307	0.177	−0.110	−0.034	0.051	0.306
	*p*		0.755	0.145	0.000 **	0.046 *	0.215	0.707	0.566	0.000 **
B. Knowledge score	*r*		-	0.224	0.830	0.028	0.210	0.942	0.197	0.184
	*p*			0.011 *	0.000 **	0.751	0.017 *	0.000 **	0.026 *	0.038 *
C. Belief score	*r*			-	0.686	−0.198	0.267	0.125	0.587	−0.013
	*p*				0.000 **	0.025 *	0.002 **	0.158	0.000 **	0.883
D. Total score	*r*				-	−0.039	0.258	0.726	0.444	0.193
	*p*					0.664	0.003 **	0.000 **	0.000 **	0.029 *
E. Environmental quality score	*r*					-	−0.543	0.071	−0.387	0.184
	*p*						0.000 **	0.423	0.000 **	0.038 *
F. The classification of hotel	*r*						-	0.211	0.264	−0.077
	*p*							0.017 *	0.003 **	0.386
G. Whether the hotel’s public appliances spread diseases	*r*							-	0.000	0.000
	*p*								1.000	1.000
H. Basic knowledge of infectious diseases	*r*								-	0.000
	*p*									1.000
I. Understanding of the routes of spreading infectious diseases	*r*									-
	*p*									

Notes: * *p* < 0.05, ** *p* < 0.01.

## Data Availability

The data presented in this study are openly available contacting corresponding Author.
